# Use of General Practitioner Telehealth Services During the COVID-19 Pandemic in Regional Victoria, Australia: Retrospective Analysis

**DOI:** 10.2196/39384

**Published:** 2023-02-07

**Authors:** Feby Savira, Liliana Orellana, Martin Hensher, Lan Gao, Andrew Sanigorski, Kevin Mc Namara, Vincent L Versace, John Szakiel, Jamie Swann, Elizabeth Manias, Anna Peeters

**Affiliations:** 1 Deakin Health Economics, Institute for Health Transformation School of Health and Social Development Deakin University Burwood Australia; 2 Global Centre for Preventive Health and Nutrition, Institute for Health Transformation School of Health and Social Development Deakin University Geelong Australia; 3 Biostatistics Unit Faculty of Health Deakin University Geelong Australia; 4 Menzies Institute for Medical Research University of Tasmania Hobart Australia; 5 Deakin Rural Health School of Medicine Warrnambool Australia; 6 Western Victoria Primary Health Network Geelong Australia; 7 Centre for Quality and Patient Safety Research, Institute for Health Transformation School of Nursing and Midwifery Deakin University Burwood Australia; 8 School of Nursing and Midwifery Faculty of Medicine, Nursing and Health Sciences Monash University Clayton Australia; 9 Institute for Health Transformation School of Health and Social Development Deakin University Burwood Australia

**Keywords:** telehealth, rural, Australia, COVID-19, eHealth, primary care, general practitioner, GP, trend, pandemic, equity, video, virtual, consultation, telephone

## Abstract

**Background:**

In March 2020, the Australian Government expanded general practitioner (GP) telehealth services in response to the COVID-19 pandemic.

**Objective:**

This study sought to assess use patterns of GP telehealth services in response to changing circumstances (before and during the COVID-19 pandemic and with or without a lockdown) in regional Victoria, Australia.

**Methods:**

We conducted a secondary analysis of monthly Medicare claims data from July 2019 to June 2021 from 140 regional GP practices in Western Victoria. The longitudinal patterns of proportion of GP telehealth consultations stratified by type of consultation (ie, videoconference vs telephone) and by geographical, consumer, and consultation characteristics were analyzed.

**Results:**

Telehealth comprised 25.8% (522,932/2,025,615) of GP consultations over the 2-year period. After the introduction of the Australian telehealth expansion policy in March 2020, there was a rapid uptake in GP telehealth services (including telephone and video services), from 0% before COVID-19 to 15% (11,854/80,922) of all consultations in March 2020, peaking at 55% (50,828/92,139) in August 2020. Thereafter, the use of telehealth declined steadily to 31% (23,941/77,344) in January 2021 and tapered off to 28% (29,263/103,798) in June 2021. Telephone services and shorter consultations were the most dominant form, and those aged 15-64 years had higher telehealth use rates than younger or older age groups. The proportion of video consultations was higher during periods with government-imposed lockdowns and higher in the most socioeconomically advantaged areas compared to less socioeconomically advantaged areas.

**Conclusions:**

Our findings support the continuation of telehealth use in rural and regional Australia post pandemic. Future policy must identify mechanisms to reduce existing equity gaps in video consultations and consider patient- and system-level implications of the dominant use of short telephone consultations.

## Introduction

Telehealth is broadly defined as remote provision of care using technologies, with or without video connection [1]. Telehealth is widely advocated as an alternative model of care to face-to-face visits to improve access, quality, and timeliness of care [2], and it may help patients who are disadvantaged by distance and disability or are reliant on caregivers. Similar to other countries in the early adoption stage (such as the United Kingdom and New Zealand) [3], use of telehealth services in Australian primary care, particularly for general practitioner (GP) services, was limited prior to COVID-19. Reasons included a lack of coverage due to geographic restrictions, limited funding, and user eligibility barriers [4], as well as poor awareness among patients regarding telehealth use [5]. Indeed, GP-specific Medicare Benefits Schedule (MBS) telehealth items were only introduced for the first time in November 2019 [6], and the uptake among patients and providers was very low (<100 consultations Australia-wide) [7].

In response to COVID-19, on March 13, 2020, the Australian Government expanded Medicare-subsidized telehealth services for all Australians to enable remote delivery of care and reduce the risk of virus transmission in the community [8]. This scheme was initially run as a “bulk-billing” scheme, meaning that patients did not incur any telehealth consultation fees, and practices claimed the consultations fees through Medicare. On April 2020, GPs were no longer required to bulk bill patients and could request patient copayments in addition to Medicare reimbursement, except for patients who met certain criteria (eg, concession card holder, children younger than 16 years, and those at high risk of COVID-19) [9]. The expansion of this subsidization led to an increased use of telehealth across Australian primary care [7], including GP services*.* Victoria had the highest COVID-19 caseload of any Australian state or territory during 2020 and 2021, which was accompanied by regulations to substantially restrict the movement of people for extended periods [10]. These circumstances provide a unique opportunity to assess the extent to which patients and providers change their use of GP telehealth services in response to changing circumstances. Furthermore, there are currently no analysis of telehealth uptake exclusively in regional areas of Australia. Therefore, the objectives of this study were to describe the number of GP consultations (including telehealth consultations) and analyze longitudinal patterns of GP telehealth consultations (by geographical, consumer, and consultation characteristics) in Western Victoria, Australia, between July 2019 and June 2021.

## Methods

### Study Design

This study was a secondary analysis of general practice MBS claims data collected for population health management and research programs [11].

### Ethics Approval

The study has been approved by Deakin University Human Ethics Advisory Group (2020-389).

### Data Source

Monthly deidentified longitudinal MBS claims data from Western Victoria Primary Health Network (PHN) Health Intelligence Unit was sourced. The Western Victoria PHN covers a population size of 617,945 (32% of regional Victoria’s population as of 2016) [12]. The data cover GP health service use from approximately 140 participating regional GP practices in Western Victoria from the Practice Aggregation Tool for the Clinical Audit Tool data extraction system. This tool enables analysis and reporting of population health data by aggregating deidentified MBS claims from many practices over time.

GP health service use data included aggregated monthly number of consultations (ie, total number of Medicare consultations items claimed for GP consultations in any of the general practices registered within the Practice Aggregation Tool for the Clinical Audit Tool system for any given month). The monthly aggregate data were stratified according to type of service (ie, face-to-face, videoconference, or telephone), gender (female or male), age groups (<15 years, 15-64 years, and 65 years, and older), whether the individual is an active or inactive patient of the clinic, and length of consultation (based on MBS item number; Table S1 in Multimedia Appendix 1). Data were available from July 2019 to June 2021.

Within Australia, much of the spatial analysis is guided by the Australian Statistical Geography Standard [13]. Australia is divided into 7 hierarchical geographic levels (in ascending order): Mesh Block, Statistical Area (SA) 1, SA2, SA3, SA4, State and Territory, and Australia [14]. Every level directly aggregates to the level above [14]. In this study, each monthly aggregate data were available at the SA2 level, which represents a community of 3000 to 25,000 usual residents. The SA2 geographical level is policy relevant, as every SA2 codes represent communities that interact together socially and economically [14]. The Western Victoria PHN data cover 60 SA2s. The SA2 level data were categorized based on the Modified Monash Model (MMM) to understand the effect of regionality and the Index of Relative Socio-Economic Advantage and Disadvantage for socioeconomic status; Table 1 presents a full explanation.

**Table 1:** Operational definitions.

Factor	Definition
The start of the pandemic	In our analysis, March 2020 was considered the start of the pandemic due to implementation of COVID-19 MBS^a^ telehealth items for the first time across Australia on March 13, 2020. Therefore, months prior to March 2020 were considered as the pre–COVID-19 period.
Lockdown periods	The Western Victoria region is located in regional Victoria, and therefore, followed regional pandemic rulings and restrictions (supplementary information 1 in Multimedia Appendix 1). Government-imposed lockdowns in regional Victoria took place in the months of April and August 2020. The months of May 2020, September 2020, February 2021, and June 2021 had at least one week period wherein lockdown was in place, and they were considered a “mixed period” (with and without lockdown).
Geographical classification	Each SA^b^2 area was categorized based on the MMM^c^ classification: MM1 (metropolitan), MM2 (regional centers), MM3 (large rural towns), MM4 (medium rural towns), and MM5 (small rural towns) [15]. MMM was chosen due to contemporary policy relevance [16,17].
Socioeconomic status classification	Each SA2 area was categorized based on SEIFA^d^ published by the ABS^e^. The chosen socioeconomic index was IRSAD^f^. It represents the socioeconomic conditions of people and households within an area and considers both relative measures of advantage and disadvantage. It has been presented into 5 quintiles, wherein 1 is the most disadvantaged and 5 is the most advantaged.
Length of consultation classification^g^	Short (attendance for obvious problem); medium (attendance <20 minutes); long (attendance for ≥20 minutes); very long (attendance for ≥40 minutes; Table S1 inMultimedia Appendix 1).
Active patients	Patients were considered active if they visited the clinic at least three times in the last 2 years; otherwise they were considered inactive. The patient visit status is valid at the time of data extraction (July 19, 2021).
Australian financial year	An Australian financial year starts on first of July and ends on the 30th of June of the following year.

^a^MBS: Medicare Benefits Schedule.

^b^SA: Statistical Area.

^c^MMM: Modified Monash Model.

^d^SEIFA: Socio-Ecocomic Indexes for Areas.

^e^ABS: Australian Bureau of Statistics.

^f^IRSAD: Index of Relative Socio-Economic Advantage and Disadvantage.

^g^Short consultations comprised claims from Medicare Benefits Schedule (MBS) items 3; 91,790; and 91,795. Medium consultations comprised claims from MBS items 23; 91,800; and 91,809. Long consultations comprised claims from MBS items 36; 92,801; and 91,810. Very long consultations comprised claims from MBS items 44; 91,802; and 91,811.

To compare the overall GP telehealth use pattern in Western Victoria to Victoria-wide data and other states in Australia (where possible), publicly available MBS data from Services Australia [18] were analyzed (supplementary information 2 in Multimedia Appendix 1). The GP telehealth use data were only available at a state level, and telehealth use data in different sociodemographic groups were not available.

### Statistical Analysis

Table 1 for contains operational definitions. We calculated the crude number of GP consultations in the Western Victoria region (including telehealth-specific consultation) over the 2-year period (July 2019 to June 2020) before and during COVID-19 and in periods with and without lockdown. Months that included a full lockdown or mixed periods were considered as a lockdown period (Table 1 and supplementary information 1 in Multimedia Appendix 1). We also determined the excess number of GP consultations during 2020-2021 financial year compared to (if any) 2019-2020 (supplementary information 3 in Multimedia Appendix 1). The monthly pattern of telehealth consultations in Western Victoria (ie, proportion of total GP consultations) stratified by mode of delivery (eg, telephone or video) was described using graphics and summary measures.

Generalised linear mixed (multilevel) models, with SA2 as the unit of observation, were used to explore whether use of GP telephone and video consultations (proportion of total GP consultation) was associated with different factors, including patient characteristics (eg, age and gender), patient status (eg, active or inactive—3 visits across a 2-year period to a GP clinic was considered active), geographical characteristics (ie, regionality and socioeconomic group), and consultation characteristics (eg, length, based on MBS item number; Table S1 in Multimedia Appendix 1). To control for clustering (ie, repeated measures), a random-effects approach was used using SA2 as the parameter. The Wald test for the interaction term was performed to assess if telephone or video use in different groups followed different patterns across time. For factors showing significant interaction, pairwise comparisons (Sidak adjusted) were performed each month. The mean percentage and 95% CIs were reported. Only data from March 2020 onward were included in the models because prior to March 2020, there was zero use of MBS-backed GP telehealth services in the region.

GP telehealth use data from Services Australia [18] were analyzed using graphics and summary measures and presented as a proportion of total GP consultations in each Australian state (Multimedia Appendix 1). Data analysis was conducted on Stata (version 17; StataCorp LLC).

## Results

### Overview of GP Consultations in Western Victoria

GP services comprised the majority (n=2,136,344, 94.8%) of primary care services in our data set (including standard GP, nurse, other medical practitioner, urgent care, and aboriginal services; Figure S1 in Multimedia Appendix 1). We also observed an increase of 9.3% (88,979/959,071) in total GP consultations during the 2020-2021 financial year compared to 2019-2020 (supplementary information 3 in Multimedia Appendix 1). March 2020 until August 2020 represents periods with significant changes, including the onset of COVID-19 pandemic, the start of telehealth expansion in primary care, and government-imposed lockdowns. During this period, face-to-face consultations decreased from 85.3% (69,068/80,922) of all consultations in March 2020 to 44.8% (41,311/92,139) in August 2020. Telehealth consultations replaced face-to-face consultations (March 2020: 11,854/80,922, 14.6%; and August 2020: 50,828/92,139, 55.2%), more than offsetting the reduction in face-to-face consultations (Figure S2 in Multimedia Appendix 1).

### Patterns In Monthly Use of GP Telehealth Consultations

**Figure 1:**
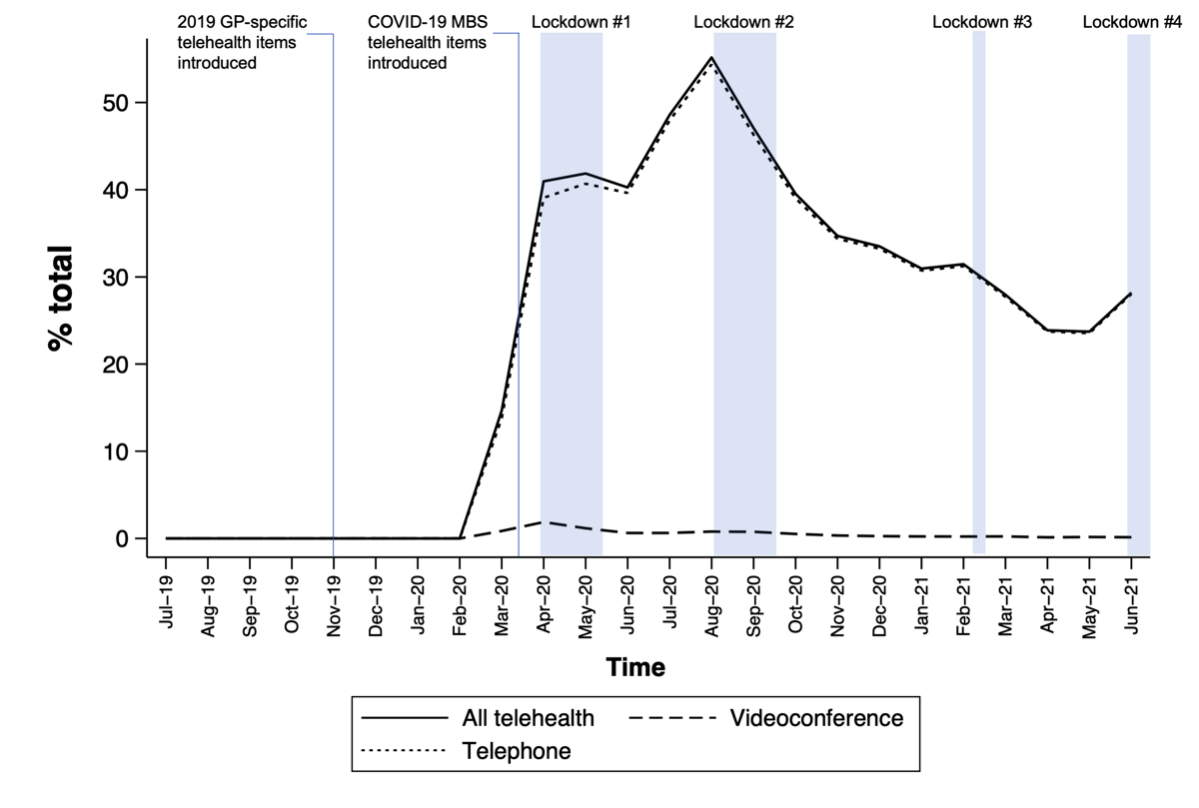
The pattern of monthly general practitioner (GP) telehealth consultations from July 2019 to June 2021 in Western Victoria, Australia.

Telehealth comprised 25.8% (522,932/2,025,615) of GP consultations from July 2019 until June 2021 (Figure 1). In March 2020, when the telehealth expansion policy was introduced, there was an immediate increase in GP telehealth use, from no telehealth usage (0%) in the region before telehealth items were introduced to 14.6% (11,854/80,922) of all consultations (Figure 1 and Figure S2 in Multimedia Appendix 1). Telehealth use further increased to 41% (36,323/86,770) in April 2020 and peaked at 55.2% (50,828/92,139) in August 2020 (coinciding with the longest statewide lockdown in Victoria). In the following months, use of telehealth steadily declined to 31% (23,941/77,344) in January 2021 and 28.2% (29,263/103,798) in June 2021 (latest available data at the time of analysis).

Telephone comprised 98.4% (n=514,565) of telehealth consultations, and its use pattern over time closely followed the overall trend for all telehealth consultations (Figure 1). The highest rate of telephone consultation was observed in August 2020 (50,102/92,139, 54.4%). Use of videoconference peaked in April 2020 at 1.9% (1,740/92,576) and remained very low throughout the pandemic (<1% since June 2020 and only 0.1%, 146/103,798 in June 2021).

During the COVID-19 pandemic (March 2020 onward), of total telehealth consultations (during lockdown: n=222,240; without lockdown: n=300,680), the proportion of video consultations was slightly higher in periods with lockdown (n=4537, 2%) compared to the periods without lockdown (n=3600, 1.2%; Figure S1 and Table S2 in Multimedia Appendix 1). Of note, the 4 lockdown periods comprised only 6 out of 16 months of the postpandemic analysis period (Table 1 offers explanation about lockdown periods).

### Patterns in GP Telehealth Consultations During COVID-19

#### Demographics

**Figure 2:**
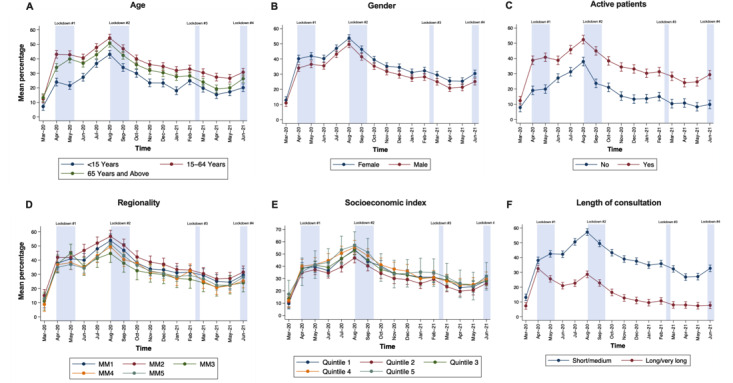
Analysis of monthly patterns of telephone consultations stratified by geographical, consumer, and clinical characteristics across 60 Statistical Areas Level 2 (SA2s) in western Victoria, Australia. A patient is considered active if they have visited the clinic at least three times in the last 2 years. For regionality—MM1: metropolitan; MM2: regional centers; MM3: large rural towns; MM4: medium rural towns; and MM5: small rural towns. For length of consultation—short: consultations for obvious problem; medium: less than 20 minutes; long: at least 20 minutes; very long: at least 40 minutes. Data are presented as mean percentage of GP telephone consultations for each month with 95% CIs.

**Figure 3:**
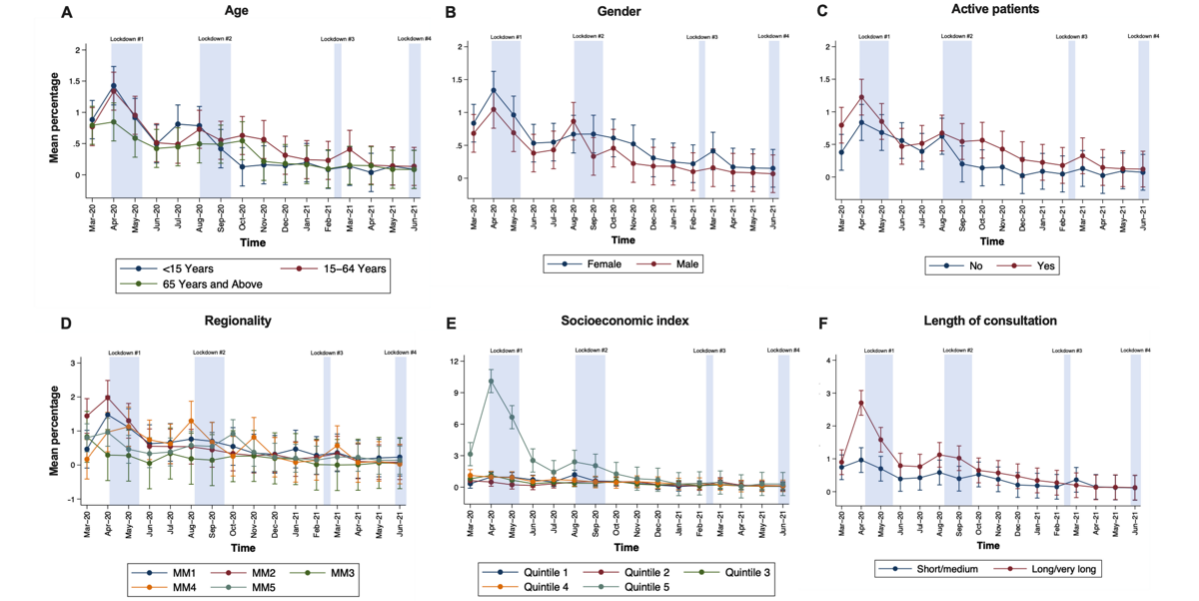
Analysis of monthly patterns of video consultations stratified by geographical, consumer, and clinical characteristics across 60 Statistical Areas Level 2 (SA2s) in Western Victoria, Australia. A patient is considered active if they have visited the clinic at least three times in the last 2 years. For regionality—MM1: metropolitan; MM2: regional centers; MM3: large rural towns; MM4: medium rural towns; and MM5: small rural towns. For length of consultation—short: consultations for obvious problem; medium: less than 20 minutes; long: at least 20 minutes; very long: at least 40 minutes. Data are presented as mean percentage of GP telephone consultations for each month with 95% CIs.

Patterns of use of GP telehealth consultations was significantly different across age groups (interaction × month; *P*<.001; Figure 2A). From March 2020 to June 2021, the uptake of telephone items was highest in the 15-64 years age group, followed by the age group ≥65 years and the age group <15 years. The difference in mean proportion of telephone use across age groups was significant for most of the time period examined. Patterns of video service uptake were similar for all age groups (Figure 3A).

#### Regionality or Rurality

The pattern of telehealth use changed with the region (interaction MMM groups × month; *P*=.001, Figure 2D). The mean percentage of telephone usage was significantly lower in the months of June, August, and September 2020 for MM3 (large rural towns) compared to MM2 (regional centers); in June and September 2020, it was lower for MM4 (medium rural towns) compared to MM2; and in June and July 2020, it was lower for MM5 (small rural towns) compared to MM2. No significant differences were observed across MMM groups for video consultations (Figure 3D).

#### Socioeconomic Status

Although no interaction between socioeconomic group and time was observed for telephone consultations (Figure 2E), the patterns of video consultations differed across the 16-month period (interaction quintile groups × month; *P*<.001; Figure 3E). Mean video use in areas categorized as quintile 5 (most advantaged) was significantly higher than quintiles 1-4 from March until June 2020. In August 2020, usage rate was also significantly higher in quintile 5 areas versus quintiles 2-4 and in quintile 1 area (most disadvantaged) versus quantile 3. In September 2020, only video use in quintile 5 was significantly higher than that of quantile 3 area. Except for March 2020, months involving significant differences coincided with government-imposed lockdown periods.

#### Length of Consultation

The pattern of telephone use was significantly different throughout the 16-month period (interaction length of consultation groups × month; *P*<.001). Short and medium-length consultations consistently accounted for a greater proportion of telephone consultation than long or very long consultations (*P*<.001 for each month; Figure 2F and Figure S3 in Multimedia Appendix 1). For the short or medium-length consultation group, the mean telephone use reached a peak in August 2020 and has since stabilized and remained above the level when it was first implemented in March 2020. For long or very long consultation group, the mean usage peaked in April 2020 and has plateaued after January 2021 to levels similar to March 2020.

The patterns of mean use of video consultations were not parallel between short or medium-length and long or very long consultation groups (interaction length of consultation groups × month; *P*<.001; Figure 3F). Compared to short or medium-length consultation group, the mean use for long or very long video consultation group was significantly higher in April, May, August, and September 2020, periods which coincide with government-imposed lockdowns.

### Comparison to Statewide Data

The overall GP use pattern observed in Western Victoria (Figure 1) and the finding of shorter consultations dominating telehealth (Figure 2F and Figure S3 in Multimedia Appendix 1) corroborate the results from state-level data (supplementary information 2 in Multimedia Appendix 1).

## Discussion

### Principal Findings

This is the first analysis of telehealth use focused on regional Australia assessing the use patterns under varying circumstances (eg, before and during the COVID-19 pandemic and with or without government-imposed lockdown). First, although the explosive increase of GP telehealth use in regional Victoria at the beginning of the pandemic (and following the expansion of GP telehealth services) aligns with urban GP [19,20] and statewide data (supplementary information 2 in Multimedia Appendix 1), our findings indicate that 16 months following the policy introduction, GP telehealth use had stabilized to comprise approximately 1 in 3 GP consultations in Western Victoria region. Second, there was an overall increase in GP visits across all types during the pandemic. It is unclear whether the increase in visits is pandemic-related (ie, more people becoming ill or seeking consultations for minor respiratory symptoms) or driven by the convenience of telehealth for patients and providers. In contrast, there was no significant increase in overall specialist activity [21] and mental health services [22] in Australia after the onset of COVID-19. Finally, our analysis provides insights into telehealth use across important geographical, consumer, and consultation characteristics, which has been identified as a major gap in the literature [2].

In addition to the significant uptake of GP telephone consultations (consistent with previous national [21,22] and international reports [23,24]), our analysis of PHN data and statewide data further adds that shorter GP consultations dominated these telephone services. Although videoconferencing encompassed only <1% of GP consultations during the pandemic, its use, particularly for longer consultations, appeared to be higher in periods with government-imposed lockdowns, as also seen in Ireland [25]. This increased use may be explained by a higher likelihood of patients needing video technology, such as those with long-standing or complex care needs or other conditions that would normally be examined in person. Future studies assessing the content, nature, and clinical factors driving telephone and video consultations are important to ensure the best patient outcomes and sustainability in the health system.

Australian GPs perceive video consultation as important [26], yet we found a very low uptake of video consultations beyond the initial phase of the pandemic. Similar observations were reported in an Australian study assessing telehealth for medication prescribing [27] and among UK doctors [28,29]. A qualitative analysis reported that Australian GPs preferred telephone consultations due to its simplicity and because the Medicare rebate was the same as video consultations [26]. Studies in the United States and Australia further suggest that clinicians and some patient groups (eg, older people) are discouraged by the complex setup process of video consultations [28,30-32]. For health issues requiring visual assessments, a study reported that GPs preferred telephone plus photographs compared to video consultation to reduce technological delays [25]. However, the main advantage of video consultations is that it allows clinicians to directly observe their patients and obtain visual and other additional cues that patients could potentially miss [28]. A US study reported that key predictors of video consultation uptake among primary and specialist care practitioners were mainly driven by availability of telehealth infrastructure, clinician preference, and biases toward video consultations [30]. Since the collection of our study data, the Australian federal government has announced discontinuation of the Medicare rebate for longer (>20 minute) telephone consultations from June 30, 2022. This policy will likely drive patients who require or prefer longer telephone consultations to switch to video consultations or visit their GP in person. However, given the current absence of investment of resources in improving video uptake in rural and disadvantaged areas for both patients and providers, it remains unclear whether this policy would improve the uptake of video consultations.

Use of video consultations was also higher in the most socioeconomically advantaged areas compared to areas of lower socioeconomic advantage, highlighting persistent inequity observed in Australia and abroad [20,33,34]. Although it has been shown that there were higher number of residents in MM1 (metropolitan area) in the highest deciles of the socioeconomic index [17], we did not observe any differences in the use of telehealth by regionality (ie, MMM). This may be driven by significant adoption of telehealth across all geographical areas due to the pandemic (wherein people were always encouraged to socially distance), as also seen overseas [35]. Overall, these findings underscore the need to ensure that any future expansion of reliance on video consultations does not widen existing gaps in access to care [5].

Our findings that telehealth remained widely used 16 months after its introduction suggests it has a valuable role in everyday practice and supports the introduction of ongoing telehealth items in December 2021 by the federal government [36]. Sustainability issues for telehealth that merit considerable further exploration include addressing the ideal ratio of telehealth to face-to-face visits for different patient groups, determining which telehealth modality is most appropriate for different clinical situations, and improving the use of video consultations. It is equally important to ensure that fee schedules are viable for general practice and avoid incentivizing certain modalities or consultation types based on reimbursement. The findings of our study also have direct relevance to other countries at similar stages of digital health adoption [3]. Future studies should explore use patterns according to clinical conditions, which was not available in our data set.

### Limitations

This study used monthly aggregate of Medicare use data, which only captures MBS-funded GP consultations and does not capture privately billed consultations. Further, unlike individual-level data, important information such as ethnicity or race, reasons for a GP visit, and clinical outcomes were not available. In the United States, those who were culturally and linguistically diverse were less likely to attend ambulatory telehealth visits [37]. Of note, the culturally and linguistically diverse communities in Western Victoria, Australia are very small [38]. Nonetheless, the literature, particularly in the Australian primary care setting, is severely lacking, and future analyses involving other patient and clinical factors are warranted. Lastly, identification of socioeconomic levels was based on the characteristics of the SA2 area or region where the GP practice was located rather than those of the patients who attended the general practices.

### Conclusions

In conclusion, telehealth continues to be integral to the overall provision of GP services in regional Victoria, Australia, 16 months since the telehealth policy expansion was first implemented. Our findings support telehealth use in the postpandemic recovery period in rural and regional Australia. Telephone services and shorter consultations were the most dominant form, and the patient and practice implications of these findings warrant further exploration. Video consultations were more used during periods with government-imposed lockdowns and were only observed in the highest socioeconomically advantaged area, highlighting the need for policy addressing disparity of availability and access to video consultations in areas of lower socioeconomic advantage.

## Appendix

Multimedia Appendix 1 Supplementary information and analyses.

## Abbreviations

GP: general practitioner
MBS: Medicare Benefits Schedule
MMM: Modified Monash Model
PHN: Primary Health Network
SA: Statistical Area
