# Supplementary material for: Use of General Practitioner Telehealth Services During the COVID-19 Pandemic in Regional Victoria, Australia: Retrospective Analysis
Source: J Med Internet Res. 2023 Feb 7;25:e39384. doi: 10.2196/39384 (PMC9907565; doi:10.2196/39384)
Supplement: Multimedia Appendix 1 [file jmir_v25i1e39384_app1.docx]

**Multimedia Appendix 1**

**Supplementary information and results**

**Supplementary Information 1. Timeline of government-imposed lockdown in regional Victoria (March 2020 – June 2021)**

Pandemic rulings and restrictions in Victoria

During the COVID-19 pandemic, government-imposed restrictions (including lockdowns) for metropolitan and regional Australia were not always the same (1). South-west Victoria follows regional pandemic rulings.

Weeks prior to government-imposed lockdown as well as after lockdown ends, there were often periods of COVID-related restrictions such as mask mandates and density limits in response to increasing number of cases in the community. Such rulings may also differ for metropolitan and regional Victoria (2).

The following outlines lockdown period in regional Victoria, Australia:

- 1^st^ Lockdown 1: 30 March 2020 – 11 May 2020, 42 days
- 2^nd^ Lockdown: 21 August 2020 – 9 Sept 2020, 19 days
- 3^rd^ Lockdown 3: 12 Feb 2021 – 17 Feb 2021, 5 days
- 4^th^ Lockdown 4: 27 May 2021 – 10 June 2021, 14 days

Regional Victoria underwent a total of 80 days in lockdown during the period covered by the longitudinal dataset.

**Supplementary Information 2. Analysis of statewide data.**

To compare the GP telehealth utilisation pattern in western Victoria to Victoria-wide data and other states in Australia, a separate analysis was conducted using publicly available MBS data from Services Australia (13). This included data from July 2019 to January 2021 specific to types of consultation (face-to-face, telehealth and telephone) in Victoria and other states, and total monthly GP consultation data in Victoria from January 2018 until December 2020. The aggregate data was available at a state-level.

Victoria was the only state to have the number of telehealth consultations exceed face-to-face consultations during the pandemic (Supplementary Figure S4). There was a higher number of GP consultations undertaken in 2020 compared to 2018 and 2019 in Victoria (Supplementary Figure S5). Breakdown by length of consultation indicated that the majority of shorter consultations were undertaken by telehealth (dominated by the 20-minute consults), while longer consultations were still mostly taking place in-person (Supplementary Figure S6).

**Supplementary Information 3. Calculation of excess GP consultations**

There were 870,092 GP consultations from an average of 124 practices contributing to the data in 2019-2020 financial year, and 1,155,523 GP consultations from an average of 150 practices contributing to the data in 2020-2021 financial year, respectively (Table S2).

Therefore, the adjusted number of consultations in 2020-2021 were calculated as follows:

$1,155,523 \div\frac{124}{150} =959,071$

The excess number of GP consultations in financial year 2020-2021 compared to 2019-2020 was calculated as follows:

$959,071 - 870,092= 88,979$

Therefore, there was an excess of approximately 88,979 (9.3%) GP consultations in western Victoria in financial year 2020-2021 versus 2019-2020.

**Supplementary tables**

**Table S1. List of relevant GP MBS items corresponding to type of modality and length of consultation**

| **Item details** | **Face-to-face item no.** | **Telehealth item no.** | **Telephone item no.** |
| --- | --- | --- | --- |
| Attendance for an obvious problem (short) | 3 | 91790 | 91795 |
| Attendance less than 20 minutes (medium) | 23 | 91800 | 91809 |
| Attendance at least 20 minutes (long) | 36 | 91801 | 91810 |
| Attendance at least 40 minutes (very long) | 44 | 91802 | 91811 |

**Table S2. Overview of crude number of GP consultations from July 2019 to June 2021 in south-west Victoria**

| **Type of consultation** | **Total number of consultations** | **By financial year** | | **By lockdown status (during COVID-19)*** | |
| --- | --- | --- | --- | --- | --- |
|  |  | 2019-2020 | 2020-2021 | During lockdown^$^ | No lockdown |
| Face-to-face | 1,502,683 | 750,330 | 752,353 | 345,896 | 630,587 |
| Old videoconference (2019) | 12 | 12 | 0 | 0 | 12 |
| COVID-19 telephone | 514,783 | 115,775 | 399,008 | 217,703 | 297,080 |
| COVID-19 videoconference | 8,137 | 3,975 | 4,162 | 4,537 | 3,600 |
| Total | 2,025,615 | 870,092 | 1,155,523 | 568,136 | 1,457,467 |

*From March 2020 onwards.

^$^Includes months in which both lockdown and non-lockdown periods were present in regional Victoria (May 2020, September 2020, February 2021 and May 2021).

522,932/2,025,615

**Supplementary figures**

**Figure S1. Proportion of GP consultations undertaken from July 2019 to June 2021 in south-west Victoria**

Non-GP services in this analysis included standard face-to-face, telephone and video consultation data based on MBS items for GP (as per Table S1), nurse, other medical practitioner, urgent care, and aboriginal services (data not shown).

**Figure S2. The crude number of GP telehealth consultations from July 2019 to June 2021 in south-west Victoria**

**Figure S3. Analysis of monthly patterns of telephone consultations stratified by length of consultation**

**% mean consultation**

*Short*: consultations for obvious problem; *medium*: less than 20 minutes; *long*: at least 20 minutes; *very long*: at least 40 minutes.

Proportion of short telehealth consultations were lower than medium telehealth consultations in April-May 2020 and in May 2021 in western Victoria. These periods coincided with flu vaccination campaigns (3, 4) and further demonstrated in a graphical analysis of short consultation items below.

**Figure S4. Proportion of GP telehealth consultations across Australian states from March 2020 to January 2021**

**Figure S5. Comparison of total GP consultations from 2018 to 2020 in Victoria, Australia**

**Figures S6. Proportion of GP telehealth consultations stratified by length of consultation in Victoria, Australia**

**References**

1. Lockdown Across Regional Victoria To Keep Us Safe Victoria, Australia: Premier of Victoria; 2021 [updated 12 Aug. Available from: <https://www.premier.vic.gov.au/lockdown-across-regional-victoria-keep-us-safe>.

2. Coronavirus Restrictions To Lift For Regional Victoria 2021 [updated 8 Sep. Available from: <https://www.premier.vic.gov.au/coronavirus-restrictions-lift-regional-victoria-0>.

3. Clinical update: 2020 seasonal influenza vaccines – early advice for vaccination providers [press release]. Canberra, ACT, Australia: Australian Government Department of Health2020.

4. TGA. 2021 seasonal influenza vaccines: Department of Health Therapeutic Goods Administration; 2021 [updated 12 Apr. Available from: <https://www.tga.gov.au/alert/2021-seasonal-influenza-vaccines>.
